# Association of serum Netrin-1, NSE, and S100β with brain injury severity and prognosis in patients with sepsis-associated encephalopathy

**DOI:** 10.17305/bb.2025.12215

**Published:** 2025-07-26

**Authors:** Bo Zhang, Qiong Wu, Jing Wu

**Affiliations:** 1 Department of Neurosurgery, Shijiazhuang People’s Hospital, Shijiazhuang, China; 2 Department of Neurosurgery, Hebei Medical University Second Hospital, Shijiazhuang, China; 3 Department of Function, Hebei Provincial Traditional Chinese Medicine Hospital, Shijiazhuang, China

**Keywords:** Sepsis-associated encephalopathy, SAE, Netrin-1, neuron-specific enolase, NSE, S100β, APACHE-II score, sequential/sepsis-related organ failure assessment score, Glasgow Coma Scale score, prognosis

## Abstract

Sepsis-associated encephalopathy (SAE) represents the most prevalent neurological complication of sepsis and is frequently linked to unfavorable patient outcomes. This study aimed to evaluate the prognostic significance of serum neuron-targeting axon guidance factor-1 (Netrin-1), neuron-specific enolase (NSE), and S100β levels in patients diagnosed with SAE. A retrospective analysis was performed on 120 SAE patients, measuring serum levels of Netrin-1, NSE, and S100β and correlating these with Acute Physiology and Chronic Health Evaluation-II (APACHE-II) scores. Independent risk factors for short-term mortality were identified, and the predictive values of these biomarkers were assessed both individually and in combination. Kaplan–Meier analysis was utilized to compare short-term mortality based on biomarker levels. Netrin-1 was found to be significantly downregulated, while NSE and S100β levels were upregulated in SAE patients. Lower levels of Netrin-1, alongside higher levels of NSE and S100β, correlated with elevated APACHE-II scores and increased short-term mortality. Multivariate analysis confirmed that all three biomarkers serve as independent predictors of short-term mortality. The combined assessment of Netrin-1, NSE, and S100β demonstrated superior prognostic value compared to individual biomarker. Therefore, serum levels of Netrin-1, NSE, and S100β are closely associated with the severity of brain injury in SAE and serve as effective predictors of short-term mortality, enhancing prognostic accuracy in clinical practice.

## Introduction

Sepsis is a life-threatening systemic condition caused by a dysregulated host response to infection, representing a major global health challenge responsible for approximately 11 million deaths annually [1]. Notably, 30% to 70% of sepsis patients develop sepsis-associated encephalopathy (SAE), a serious complication marked by acute brain dysfunction [2]. SAE manifests with symptoms such as altered consciousness, delirium, and, in severe cases, motor stiffness [3, 4]. It contributes to various degrees of neuronal damage and often results in long-term cognitive impairment, being closely linked to increased mortality and morbidity [5]. Importantly, patients with SAE exhibit significantly higher short-term mortality rates compared to those with sepsis alone [3, 4], underscoring the critical need for early diagnosis, severity assessment, and accurate prognostic evaluation.

The pathogenesis of SAE primarily involves systemic inflammation and cerebral perfusion abnormalities, both of which disrupt brain homeostasis and function [6]. Inflammatory mediators accumulating in the brain impair cellular metabolism and neurophysiological processes, contributing to neuronal injury. Among potential biomarkers, neuron-targeting axon guidance factor-1 (Netrin-1), a soluble protein involved in axonal guidance, has gained attention. Widely expressed in the nervous system and peripheral organs, Netrin-1 exhibits anti-inflammatory properties through its interaction with macrophage surface receptors such as UNC5B (uncoordinated-5 homolog B) [7, 8]. Notably, decreased Netrin-1 levels have been observed in patients with ischemic cerebrovascular disease [9], and exogenous Netrin-1 administration has been shown to enhance neurological recovery and maintain blood–brain barrier (BBB) integrity in rat models of middle cerebral artery occlusion [10]. Additionally, dynamic changes in serum Netrin-1 levels have been linked to short-term prognosis and neurological outcomes [11]. While Netrin-1 has been widely studied in cancer and angiogenesis-related disorders [9, 12, 13], and more recently in sepsis-associated acute kidney and lung injuries [14, 15], its role in SAE remains underexplored.

In addition to Netrin-1, other key biomarkers have shown promise in reflecting neurological injury in systemic inflammatory conditions like SAE. Neuron-specific enolase (NSE), a sensitive marker of neuronal damage, is notably elevated in various central nervous system (CNS) pathologies, including traumatic brain injuries, polyneuropathy, and brain injury syndromes. Elevated serum NSE levels are often inversely associated with patient prognosis in brain injury-related diseases [16]. During brain injury, neuronal disintegration and BBB breakdown facilitate the release of NSE into the bloodstream and cerebrospinal fluid [17]. Clinically, NSE is recognized as an important indicator for assessing CNS damage severity [18].

Another vital biomarker is specific protein β (S100β), a calcium-binding protein abundantly expressed in glial cells and certain neurons, particularly in the cerebellum and brainstem [19]. S100β plays diverse roles in neuronal differentiation, proliferation, and apoptosis [20]. Depending on its concentration, S100β can exert neurotoxic or neurotrophic effects [21]. At low concentrations, it supports neuronal repair and regeneration, whereas at high concentrations, it may impair neuronal function through mechanisms such as nitric oxide-mediated neurotoxicity [22]. Therefore, the differential expression of S100β holds clinical relevance in diagnosing and monitoring brain injury severity.

Despite individual studies exploring these biomarkers in neurological or septic conditions, few have comprehensively assessed their combined prognostic utility in SAE. Thus, this study aimed to investigate the correlation between serum Netrin-1, NSE, and S100β levels with the severity of brain injury and 28-day prognosis in SAE patients, potentially offering a novel multi-marker strategy for early diagnosis and risk stratification in SAE.

## Materials and methods

### Sample size estimation

Sample size was calculated using G*Power 3.0.10 software (Heinrich-Heine-Universität Düsseldorf, Germany). An independent samples *t*-test was selected as the statistical test. Parameters were set as follows: *α* ═ 0.05, *β* ═ 0.95, and an effect size of 0.5, with all *P* values two-sided. The resulting minimum estimated sample size required was 210 patients (Figure S1).

### Study population

This retrospective study enrolled septic patients admitted to Shijiazhuang People’s Hospital between May 2022 and May 2023. A total of 330 patients were initially screened. After applying the inclusion and exclusion criteria, 310 patients were deemed eligible. However, 25 were excluded based on exclusion criteria, 13 declined to participate, and 12 withdrew during the study. Ultimately, 260 septic patients were included.

Patients were categorized into two groups: the SAE group (*n* ═ 120), consisting of sepsis patients with SAE, and the N-SAE group (*n* ═ 140), comprising sepsis patients without encephalopathy. SAE diagnosis was made according to clinical and neurological assessment standards.

For prognostic evaluation, patients in the SAE group were further stratified based on their 28-day survival status: the survival subgroup (*n* ═ 80) and the death subgroup (*n* ═ 40). Short-term prognosis was defined as survival within 28 days of admission.

### Primary and secondary outcomes

The primary outcomes were the correlations of serum Netrin-1, NSE, and S100β levels with the severity of brain damage in SAE, and the predictive value of these markers for their short-term (28-day) mortality in SAE patients.

Secondary analyses included correlations between Netrin-1 and BBB damage-related biomarkers NSE and S100β, as well as correlations of Netrin-1, NSE, and S100β with systemic inflammatory markers in SAE patients.

### Inclusion and exclusion criteria

Inclusion criteria were as follows: (1) Patients met the diagnostic criteria for Sepsis-3 [23]: defined as life-threatening organ dysfunction caused by a dysregulated host response to infection, with a Sequential/Sepsis-related Organ Failure Assessment (SOFA) score ≥ 2; (2) Diagnosed with sepsis and admitted to the intensive care unit (ICU) for treatment; (3) Aged 18–85 years; (4) Possessed complete clinical data, including medical history, laboratory results, and treatment records; (5) Had no co-existing infectious disorders such as tuberculosis, HIV/AIDS, or hepatitis B.

Exclusion criteria were as follows: (1) History of chronic drug or alcohol abuse; (2) Presence of severe metabolic disorders; (3) Renal or hepatic failure, or malignancy; (4) Major burns; (5) Prior history of neurological diseases; (6) Immune system diseases or long-term immunosuppressive therapy; (7) Pregnant or lactating women; (8) Incomplete clinical documentation.

Diagnostic criteria for SAE: Given the absence of unified diagnostic guidelines for SAE, this study adopted established criteria from prior literature [24–26]. Patients were required to meet sepsis diagnostic criteria alongside evidence of CNS dysfunction. CNS abnormalities included acute or subacute changes in consciousness, disorientation, cognitive impairment, or delirium, as assessed by the confusion assessment method for the ICU (CAM-ICU). Intracranial infections and other acute CNS pathologies were excluded via electroencephalography (EEG), head computed tomography (CT), and magnetic resonance imaging (MRI).

### Data and sample collection

Clinical baseline data were extracted from electronic medical records and included the following parameters: age at admission, body mass index (BMI), sex, systolic blood pressure (SBP), average heart rate, diastolic blood pressure (DBP), partial pressure of carbon dioxide (PCO_2_), blood oxygen saturation (SPO_2_), partial pressure of oxygen (PO_2_), interleukin-6 (IL-6), interleukin-10 (IL-10), C-reactive protein (CRP), serum creatinine (Scr), blood urea nitrogen (BUN), aspartate aminotransferase (AST), alanine aminotransferase (ALT), growth hormone-releasing peptide (Ghrelin), mean cerebral blood flow velocity (Vm), and peak systolic velocity (Vs). Additionally, severity assessments were recorded using three clinical scoring systems: the Acute Physiology and Chronic Health Evaluation Scoring System-II (APACHE-II), SOFA, and the Glasgow Coma Scale (GCS).

Peripheral venous blood samples (4 mL) were collected from all participants in a fasting state within 48 h of admission. These samples were subsequently analyzed for serum levels of Netrin-1, NSE, and S100β using enzyme-linked immunosorbent assay (ELISA).

The APACHE-II scale was utilized to evaluate the severity of brain injury in SAE patients. Scores range from 0 to 71, with higher scores indicating more severe physiological derangement. Based on APACHE-II scores, patients were stratified into three risk categories: low-risk group (< 10 points), moderate-risk group (10–20 points), and high-risk group (> 20 points).

The SOFA score, which assesses dysfunction in six organ systems, including respiratory, hepatic, coagulation, neurological, circulatory, and renal, ranges from 0–24 points, with higher scores reflecting increased disease severity.

The GCS score, ranging from 3 to 15, was used to assess the level of consciousness: a score > 8 indicates a better prognosis, a score < 7 suggests a poor outcome, and scores between 3 and 5, in combination with absent brainstem reflexes, suggest a high risk of mortality.

### ELISA

Serum concentrations of S100β, NSE, and Netrin-1 were quantified using ELISA. The assays were conducted in strict accordance with the manufacturer’s protocols: S100β kit (XY2455A, XYBIO), NSE kit (FY-03237H2, FUYUBIO), and Netrin-1 kit (KBH1277, Krishgen Biosystems, Mumbai, India).

### Ethical statement

This retrospective study was conducted in accordance with the ethical principles outlined in the *Declaration of Helsinki* and relevant national clinical research regulations. The protocol adhered to the standards recommended by the Enhancing the QUAlity and Transparency Of health Research (EQUATOR) network guidelines. Ethical approval was granted by the Academic Ethics Committee of Shijiazhuang People’s Hospital (No.: SH-2022-0302).

### Statistical analysis

Data were analyzed using SPSS version 21.0 (IBM Corp., Armonk, NY, USA), MedCalc version 19.0 (MedCalc Software Ltd., Ostend, Belgium), and GraphPad Prism version 8.0.1 (GraphPad Software Inc., San Diego, CA, USA). Normality of continuous variables was evaluated using the Kolmogorov–Smirnov test. Normally distributed data were expressed as mean ± standard deviation. Comparisons between two groups were performed using the independent sample *t-*test. Non-normally distributed data were expressed as median with interquartile range (IQR). The Mann–Whitney *U* test was used for two-group comparisons. Categorical variables were summarized as counts and percentages, with inter-group differences assessed via the chi-square (χ^2^) test. Correlation analyses were performed using Pearson’s correlation coefficient, including correlations between Netrin-1 and BBB injury markers (NSE and S100β), and associations of Netrin-1, NSE, and S100β levels with inflammatory markers and APACHE-II score in SAE patients.

Logistic regression analysis was used to identify independent risk factors for 28-day mortality. Odds ratios (ORs) with 95% confidence intervals (CIs) were calculated. Receiver operating characteristic (ROC) curves were generated to evaluate the predictive value of Netrin-1, NSE, and S100β, both individually and in combination, for short-term mortality in SAE patients. The DeLong test was used for comparison of AUC values; a Bonferroni-corrected *α* ═ 0.017 was used for pairwise comparisons. Kaplan–Meier survival curves were constructed to assess cumulative 28-day mortality in relation to biomarker levels. Group differences were tested using the log-rank test with *α* ═ 0.05. All *P* values were two-sided, with *P* < 0.05 considered statistically significant unless otherwise specified.

## Results

### Baseline characteristics of patients

Clinical data were collected from a total of 260 patients diagnosed with sepsis (Table 1). Among them, 120 patients met the criteria for SAE and were categorized as the SAE group, while the remaining 140 patients without neurological complications were included in the non-SAE (N-SAE) group.

**Table 1:** Baseline information of the enrolled patients

	**SAE (*n* ═ 120)**	**N-SAE (*n* ═ 140)**	***P***	**Non-survivor subgroup (*n* ═ 40)**	**Survivor subgroup (*n* ═ 80)**	***P***
Age (years)	59.33 ± 9.11	58.29 ± 8.14	0.332	60.13 ± 8.03	58.93 ± 9.62	0.503
Sex (Male/Female)	64/56	89/51	0.102	22/18	42/38	0.848
BMI (kg/m^2^)	22.97 ± 1.43	22.73 ± 1.21	0.140	23.04 ± 1.31	22.94 ± 1.49	0.712
Diabetes (*n*, %)	27 (22.5%)	24 (17.14%)	0.347	13 (32.50%)	14 (17.50%)	0.103
Hypertension (*n*, %)	52 (43.33%)	51 (36.43%)	0.309	21 (52.50%)	31 (38.75%)	0.174
Average heart rate (beats/min)	81.28 ± 10.53	79.76 ± 9.06	0.212	81.466 ± 9.75	81.19 ± 10.96	0.895
SBP (mmHg)	110.50 ± 14.33	108.36 ± 13.83	0.221	110.65 ± 14.49	110.20 ± 14.18	0.871
DBP (mmHg)	70.38 ± 6.34	69.31 ± 5.32	0.138	70.65 ± 6.61	70.25 ± 6.24	0.745
SPO_2_ (%)	94.49 ± 3.13	97.41 ± 2.29	<0.001	93.70 ± 3.23	94.89 ± 3.03	0.048
PCO_2_ (mmHg)	40.70 ± 4.65	40.21 ± 3.76	0.349	40.92 ± 4.73	40.59 ± 4.63	0.717
PO_2_ (mmHg)	111.58 ± 9.57	110.17 ± 8.43	0.209	111.33 ± 9.16	111.7 ± 9.75	0.843
APACHE-II score	15 (3, 42)	8 (2, 15)	<0.001	22 (12, 42)	13 (3, 19)	<0.001
SOFA score	11 (2, 22)	7 (2, 11)	<0.001	15 (5, 22)	9 (2, 17)	<0.001
GCS score	12 (5, 15)	13 (9, 15)	<0.001	11.5 (7, 15)	12 (5, 15)	0.049
IL-6 (pg/mL)	249.20 ± 58.76	221.37 ± 41.62	<0.001	305.67 ± 54.99	220.97 ± 35.88	<0.001
IL-10 (pg/L)	130.39 ± 36.23	122.27 ± 28.99	0.046	138.49 ± 39.48	126.33 ± 34.03	0.083
CRP (mg/L)	17.52 ± 4.41	16.48 ± 3.82	0.044	18.05 ± 4.97	17.26 ± 4.12	0.357
Scr (mg/dL)	1.09 ± 0.13	0.94 ± 0.11	<0.001	1.08 ± 0.13	1.1 ± 0.14	0.461
BUN (mg/dL)	22.27 ± 3.73	19.654 ± 3.557	<0.001	21.69 ± 3.76	22.56 ± 3.7	0.226
AST (U/L)	31.39 ± 6.27	30.67 ± 5.33	0.321	30.78 ± 5.91	31.69 ± 6.45	0.460
ALT (U/L)	41.40 ± 5.47	40.66 ± 4.83	0.245	40.81 ± 5.26	41.7 ± 5.59	0.404
Ghrelin (mg/mL)	815.67 ± 73.92	523.77 ± 41.32	<0.001	893.14 ± 62.22	777.23 ± 42.23	<0.001
Vm (cm/s)	144.68 (68.27, 221.74)	129.03 (83.63, 189.69)	<0.001	145.87 (59.12, 133.34)	144.33 (41.11, 137.2)	0.048
Vs (cm/s)	88.73 (41.11, 137.2)	82.04 (41.39, 121.91)	<0.001	91.95 (59.12, 137.20)	85.03 (41.11, 116.20)	0.049

Measurement data following normal distribution were denoted as mean ± standard deviation. For inter-group comparisons, the independent sample *t*-test was implemented. Non-normally distributed measurement data were expressed as quartiles, with inter-group comparisons made by the Mann–Whitney *U* test. Count data were presented as percentage (%), with inter-group comparisons conducted by the chi-square test. BMI: Body mass index; SBP: Systolic blood pressure; DBP: Diastolic blood pressure; SPO_2_: Oxygen saturation; PCO_2_: Partial pressure of carbon dioxide; PO_2_: Partial pressure of oxygen; APACHE-II: Acute Physiology and Chronic Health disease Classification System II; SOFA: Sequential/sepsis-related organ failure assessment; GCS: Glasgow Coma Scale; IL: Interleukin; CRP: C-reactive protein; Scr: Serum creatinine; BUN: Blood urea nitrogen; AST: Aspartate aminotransferase; ALT: Alanine aminotransferase; Ghrelin: Growth hormone-releasing peptide; Vm: Mean blood flow velocity; Vs: Peak systolic blood flow velocity; SAE: Sepsis-associated encephalopathy; N-SAE: Non-Sepsis-associated encephalopathy.

No statistically significant differences were observed between the two groups in terms of age, sex, BMI, SBP, DBP, average heart rate, PCO_2_, PO_2_, AST, or ALT (all *P* > 0.05). However, the SAE group demonstrated significantly higher APACHE-II and SOFA scores, as well as increased serum levels of IL-6, IL-10, CRP, Scr, BUN, and Ghrelin, and decreased SpO_2_, Vs, and Vm compared to the N-SAE group (all *P* < 0.05).

Within the SAE cohort, patients were further subdivided based on 28-day survival status into a survivor subgroup (*n* ═ 80) and a non-survivor subgroup (*n* ═ 40). No significant differences were found in age, sex, BMI, average heart rate, SBP, DBP, PCO_2_, PO_2_, IL-10, CRP, Scr, BUN, AST, or ALT between these two subgroups (all *P* > 0.05). However, SPO_2_, APACHE-II score, SOFA score, IL-6, Ghrelin, Vm, and Vs significantly differed between survivors and non-survivors (all *P* < 0.05).

### Differential expression of Netrin-1, NSE, and S100β in SAE patients

**Figure 1.**
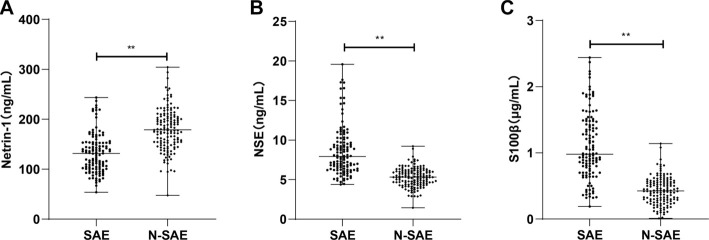
**Serum expression levels of Netrin-1, NSE, and S100β in patients with SAE and N-SAE.** Comparison of serum levels of Netrin-1 (A), NSE (B), and S100β (C) between SAE and N-SAE patients. Data are presented as medians and interquartile ranges. Inter-group differences were assessed using the Mann–Whitney *U* test. **P < 0.01. Netrin-1: Neuron-targeting axon guidance factor-1; NSE: Neuron-specific enolase.

Serum levels of Netrin-1, NSE, and S100β were quantified using ELISA. Compared to N-SAE patients, SAE patients exhibited significantly lower levels of Netrin-1, along with elevated serum levels of NSE and S100β (Figure 1A–1C, all *P* < 0.01). These findings suggest that Netrin-1 is downregulated, while NSE and S100β are upregulated in SAE, potentially reflecting the extent of neuronal injury.

### Correlation of biomarker expression with brain injury severity in SAE

The severity of brain injury among SAE patients was stratified using the APACHE-II scoring system, yielding 27 patients in the low-risk group (score < 10), 68 in the moderate-risk group (10–20), and 25 in the high-risk group (> 20).

**Figure 2.**
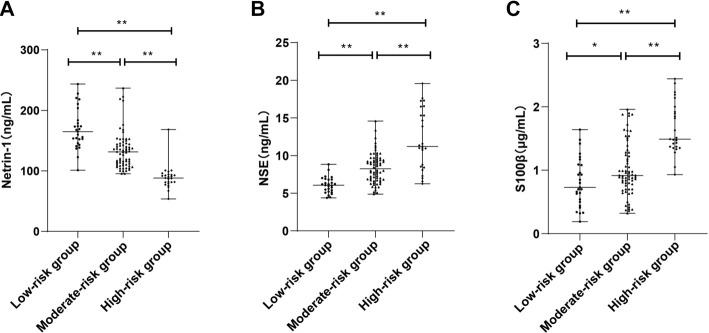
**Expression levels of Netrin-1, NSE, and S100β in SAE patients stratified by brain injury severity.** Serum concentrations of Netrin-1 (A), NSE (B), and S100β (C) in SAE patients classified into low-, moderate-, and high-risk groups based on APACHE-II scores. Non-normally distributed variables were analyzed using the Kruskal–Wallis rank-sum test with Tukey’s post hoc comparisons. **P < 0.01. Netrin-1: Neuron towards axon guidance factor-1; NSE: Neuron-specific enolase.

Analysis revealed a progressive decrease in serum NSE levels with increasing severity of brain injury (Figure 2A), while serum levels of NSE and S100β increased proportionally with disease severity (Figure 2B and 2C) (all *P* < 0.01). These results demonstrate that expression patterns of Netrin-1, NSE, and S100β correlate significantly with the degree of neurological dysfunction in SAE.

### Serum Netrin-1 is negatively correlated with BBB injury markers in SAE patients

Given that NSE and S100β are established biomarkers associated with BBB disruption [27–29], we examined the relationship between serum Netrin-1 levels and these BBB-associated indicators in SAE patients. Pearson correlation analysis revealed that Netrin-1 was significantly negatively correlated with both NSE (*r* ═ –0.653, *P* < 0.05) and S100β (*r* ═ –0.460, *P* < 0.05) (Table 2), suggesting that decreased serum Netrin-1 is associated with increased BBB injury in SAE.

**Table 2:** Netrin-1 in SAE patients is significantly negatively correlated with BBB injury

**Parameter**	**NSE**		**S100β**	
	***r***	***P***	***r***	***P***
Netrin-1	−0.653	< 0.001	−0.46	< 0.001

Netrin-1: Neuron towards axon guidance factor-1; S100β: Central nervous system specific protein.

### Netrin-1, NSE, and S100β correlate with disease severity and inflammatory cytokines in SAE

To assess associations between biomarker levels and disease severity, we further performed Pearson’s correlation analyses between APACHE-II scores and levels of Netrin-1, NSE, and S100β in SAE patients. As shown in Table 3, Netrin-1 was significantly negatively correlated with APACHE-II scores (*r* ═ –0.714) as well as pro-inflammatory markers IL-6 (*r* ═ –0.633), IL-10 (*r* ═ –0.258), and CRP (*r* ═ –0.269) (all *P* < 0.05). In contrast, both NSE and S100β were positively correlated with APACHE-II scores (*r* ═ 0.795 and *r* ═ 0.666, respectively), IL-6 (*r* ═ 0.638 and *r* ═ 0.569), CRP (*r* ═ 0.421 and *r* ═ 0.412), and IL-10 (*r* ═ 0.251 and *r* ═ 0.326) (all *P* < 0.05). These results indicate that lower Netrin-1 and higher NSE and S100β levels are associated with greater disease severity and inflammatory response in SAE.

**Table 3:** S100β, Netrin-1, and NSE are correlated prominently with inflammatory factors and APACHE-II scores in SAE patients

**Parameter**	**APACHE-II score**		**IL-6**		**IL-10**		**CRP**	
	***r***	***P***	***r***	***P***	***r***	***P***	***r***	***P***
Netrin-1	−0.714	<0.001	−0.633	<0.001	−0.258	0.005	−0.269	0.003
NSE	0.795	<0.001	0.638	<0.001	0.421	<0.001	0.251	0.006
S100β	0.666	<0.001	0.569	<0.001	0.412	<0.001	0.326	<0.001

Netrin-1: Neuron towards axon guidance factor-1; NSE: Neuron-specific enolase; S100β: Central nervous system specific protein; APACHE-II: Acute Physiology and Chronic Health disease Classification System II; IL: Interleukin; CRP: C-reactive protein.

### Netrin-1, NSE, and S100β are independent predictors of 28-day mortality in SAE patients

Among the 120 SAE patients, 40 died within 28 days of admission. To identify predictors of short-term mortality, we first performed univariate logistic regression, identifying several significant factors: APACHE-II score, SOFA score, IL-6, Ghrelin, Netrin-1, NSE, and S100β. These variables were included in a multivariate logistic regression analysis, which revealed four independent predictors of 28-day mortality (Table 4): Netrin-1 (*P* ═ 0.017, OR = 0.941, 95%CI = 0.895–0.989), NSE (*P* ═ 0.015, OR = 3.349, 95%CI = 1.260–8.903), S100β (*P* ═ 0.041, OR = 57.760, 95%CI = 1.172–2846.201), and Ghrelin (*P* ═ 0.031, OR = 1.063, 95%CI = 1.006–1.124). Thus, NSE and S100β emerged as risk factors, while Netrin-1 acted as a protective factor for short-term mortality in SAE.

**Table 4:** NSE, Netrin-1, and S100β were risk factors for 28-day mortality of SAE patients

	**Univariate analysis**			**Multivariate analysis**		
	***P***	**OR**	**95% CI**	***P***	**OR**	**95% CI**
SPO_2_	0.052	0.879	0.771–1.001	–	–	–
APACHE-II score	<0.001	1.575	1.305–1.902	0.266	1.316	0.811–2.134
SOFA score	<0.001	1.444	1.256–1.660	0.281	0.786	0.507–1.218
GCS score	0.051	0.859	0.737–1.001	–	–	–
IL-6	<0.001	1.045	1.028–1.063	0.738	0.994	0.957–1.031
Ghrelin	<0.001	1.062	1.037–1.089	0.031	1.063	1.006–1.124
Vm	0.052	1.016	1.000–1.033	-	-	-
Vs	0.054	1.026	1.000–1053	-	-	-
Netrin-1	<0.001	0.918	0.890–0.948	0.017	0.941	0.895–0.989
NSE	<0.001	3.094	2.008–4.767	0.015	3.349	1.260–8.903
S100β	<0.001	39.109	10.372–147.461	0.041	57.760	1.172–2846.201

SPO_2_: Oxygen saturation; PCO_2_: Partial pressure of carbon dioxide; PO_2_: Partial pressure of oxygen; APACHE-II: Acute Physiology and Chronic Health disease Classification System II; SOFA: Sequential/Sepsis-related Organ Failure Assessment; GCS: Glasgow Coma Scale; IL: Interleukin; Ghrelin: Growth hormone-releasing peptide; Vm: Mean blood flow velocity; Vs: Peak systolic blood flow velocity; Netrin-1: Neuron towards axon guidance factor-1; NSE: Neuron-specific enolase; S100β: Central nervous system specific protein.

### Prognostic performance of Netrin-1, NSE, and S100β for 28-day mortality in SAE

**Figure 3.**
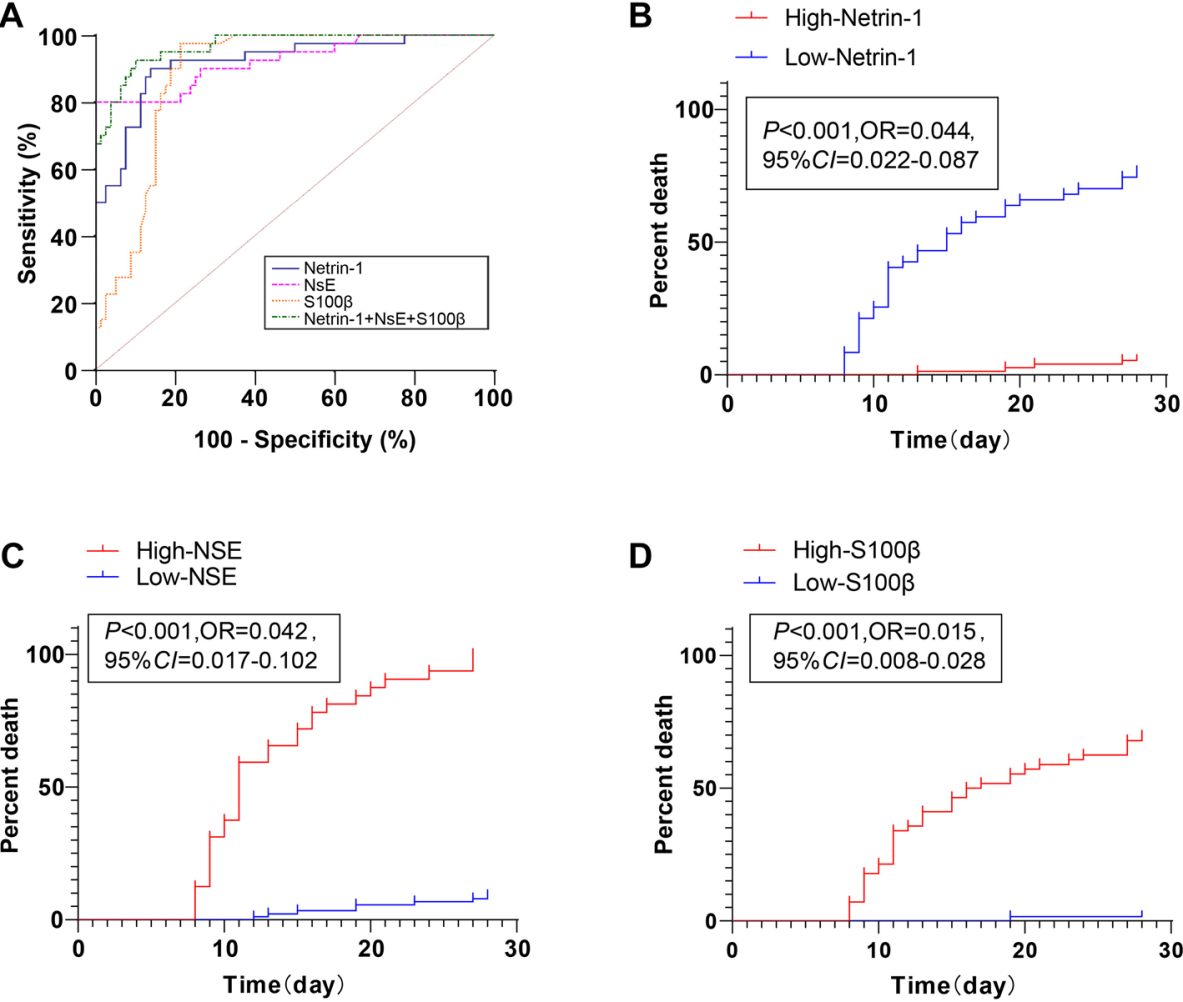
**Predictive value of Netrin-1, NSE, S100β, and their combination detection for 28-day mortality in SAE patients.** (A) ROC curves showing the predictive performance of Netrin-1, NSE, S100β, and their combination for short-term (28-day) mortality in SAE patients; (B–D) Kaplan–Meier survival curves for short-term mortality based on expression levels of Netrin-1 (B), NSE (C), and S100β (D). Patients with low Netrin-1, high NSE, or high S100β levels demonstrated significantly poorer survival, as reflected by leftward shifts in the curves. Netrin-1: Neuron towards axon guidance factor-1; NSE: Neuron-specific enolase.

To evaluate their prognostic utility, we conducted ROC curve analysis for serum Netrin-1, NSE, and S100β (Figure 3A): Netrin-1 (area under the curve [AUC] ═ 0.919, cutoff = 114.40, Sensitivity = 90.00%, Specificity = 86.25%), NSE (AUC = 0.923, cutoff = 9.66, Sensitivity = 80.00%, Specificity = 90.00%), and S100β (AUC = 0.886, cutoff = 0.99, Sensitivity = 97.50%, Specificity = 78.70%).

To explore the impact of biomarker levels on mortality, patients were stratified based on these thresholds. Kaplan–Meier survival analysis revealed that the Low-Netrin-1 group had significantly lower 28-day survival than the High-Netrin-1 group (Figure 3B). The High-NSE group had poorer survival outcomes compared to the Low-NSE group (Figure 3C). The High-S100β group also showed reduced survival relative to the Low-S100β group (Figure 3D).

Finally, combined detection of Netrin-1, NSE, and S100β yielded superior predictive power for short-term death compared to any single marker alone (Table 5), as confirmed by DeLong’s test in MEDCALC software (all *P* < 0.05). These results suggest that low Netrin-1 and elevated NSE and S100β levels are associated with increased disease severity and short-term mortality in SAE patients. Combined detection of these biomarkers offers improved predictive accuracy for 28-day mortality and could aid in early risk stratification and management.

**Table 5:** The predictive value of Netrin-1, NSE, S100β, and their combined test for 28-day mortality in SAE patients

**Items**	**Sensitivity**	**Specificity**	**AUC**	***P***	**95% CI**
Netrin-1	90.00	86.25	0.919	<0.001	0.855–0.961
NSE	80.00	90.00	0.923	<0.001	0.860–0.964
S100β	97.50	78.70	0.886	<0.001	0.815–0.937
Netrin-1 + NSE + S100β	95.0	92.50	0.983	<0.001	0.941–0.998
Netrin-1∼Netrin-1 + NSE + S100β	*P* ═ 0.008	95% CI: −0.016 to 0.111			
NSE∼Netrin-1 + NSE + S100β	*P* ═ 0.016	95% CI: 0.011–0.109			
S100Δ ∼Netrin-1 + NSE + S100β	*P* < 0.001	95% CI: 0.042–0.152			

The area under multiple ROC curves (AUC) was compared using the Delong test in MEDCalc software, and significance threshold was *a* ═ 0.017 after Bonferroni correction. *P* values were two-sided, and *P* < 0.05 or *P* < 0.017 was perceived as a statistically significant difference. Netrin-1: Neuron towards axon guidance factor-1; NSE: Neuron-specific enolase; S100β: Central nervous system specific protein.

## Discussion

Sepsis presents a serious global health challenge, contributing not only to high morbidity and mortality but also imposing substantial economic burdens on healthcare systems [30]. SAE, one of its most frequent neurological complications, remains underdiagnosed due to the lack of specific biomarkers. In this context, our study highlights the clinical relevance of three serum biomarkers, Netrin-1, NSE, and S100β, which demonstrated strong associations with inflammatory markers, disease severity, and short-term outcomes in SAE patients.

Consistent with prior research, we found that Netrin-1, an axon guidance protein with anti-inflammatory and BBB-stabilizing functions [9], was significantly downregulated in SAE patients. In addition to its diagnostic potential, NSE serves as a key neurocritical biomarker for evaluating and monitoring neurological damage in patients with SAE over time [31]. Similarly, S100β has been recognized as a valuable marker of asymptomatic brain injury during carotid revascularization procedures [32]. In the present study, we demonstrated that serum Netrin-1, NSE, and S100β were all significantly correlated with inflammatory markers and the severity of illness in SAE patients, and each independently predicted 28-day mortality, suggesting that these biomarkers may aid in early risk assessment. Furthermore, we observed a negative correlation between Netrin-1 and the BBB injury-associated markers NSE and S100β, reinforcing the role of Netrin-1 in neurovascular integrity.

The incidence of SAE is known to be elevated in diabetic patients [33], possibly due to chronic hyperglycemia and insulin resistance, which can exacerbate neuroinflammation, disrupt mitochondrial function in the hippocampus, and lower nerve growth factor (NGF) levels, thereby worsening neurological outcomes [34]. Some studies report a higher susceptibility to SAE in elderly septic patients with diabetes, with variability in diabetes prevalence between SAE and N-SAE groups [35]. However, our findings showed no significant difference in the incidence of diabetes and hypertension between SAE and N-SAE patients, consistent with other literature [36–38].

Netrin-1, as an axon guidance protein, plays a neuroprotective role by modulating inflammation and stabilizing the BBB [39]. Its known roles in synaptic remodeling, axonal regeneration, white matter repair, and neural stem cell migration are closely tied to its influence on neuroinflammation, cell death regulation, and angiogenesis [9]. Elevated NSE in cerebrospinal fluid or serum is a well-established marker of brain injury, validated in conditions such as hypoxic-ischemic encephalopathy, stroke, and traumatic brain injury [40, 41]. Indeed, NSE levels are higher in SAE patients compared to those with sepsis but without encephalopathy [42, 43].

S100β, when elevated extracellularly, promotes nitric oxide synthase expression in astrocytes, potentially leading to cell death and infarct expansion [44]. It is recognized as a highly sensitive biomarker for CNS damage and is particularly effective in detecting subclinical brain injury [19]. Hu et al. have confirmed elevated serum S100β levels in SAE individuals vs non-encephalopathy septic individuals [45], and others have shown its value in evaluating brain injury severity [46] and poor prognoses [28]. In addition, both NSE and S100β have established associations with BBB disruption [27, 29].

In alignment with this evidence, our study observed marked elevations of NSE and S100β and a decrease in Netrin-1 in the serum of SAE patients. We further demonstrated, for the first time, that serum Netrin-1 was inversely correlated with both NSE and S100β levels, suggesting a tight association between reduced Netrin-1 expression and BBB injury in SAE.

Finally, the APACHE-II score, widely regarded as a reliable predictor of SAE-related mortality, reflects the overall severity of illness [47]. Pearson’s correlation analyses confirmed a negative association of the APACHE-II score and inflammatory markers with Netrin-1, and positive correlations with NSE and S100β, underscoring the potential of these biomarkers to reflect disease burden. Nonetheless, due to constraints in time and funding, we were unable to explore the underlying molecular mechanisms linking Netrin-1 to its anti-inflammatory or BBB-stabilizing roles in SAE progression, which warrants further investigation.

Elevated serum NSE levels have consistently been associated with increased rates of poor neurological outcomes and higher mortality among SAE patients, underscoring its value as a prognostic biomarker [43]. Supporting this, Guo et al. [48] reported that higher Netrin-1 expression at admission predicted better 3-month functional recovery in patients with ischemic stroke, indicating that serum Netrin-1 may serve as a valuable prognostic biomarker for cerebrovascular outcomes. Consistent with these findings, logistic regression analysis identified Netrin-1 as a protective factor and NSE and S100β as risk factors for 28-day mortality in SAE patients.

Lower serum Netrin-1 concentrations have been independently associated with worse functional outcomes and increased mortality in various neurological disorders, including ischemic stroke, intracerebral hemorrhage, and subarachnoid hemorrhage [49]. In parallel, elevated S100β levels in sepsis patients have shown moderate correlation with SAE onset and unfavorable prognosis, reinforcing its potential as both a prognostic and diagnostic biomarker in SAE [45].

Furthermore, CRP and NSE have demonstrated significant predictive value for early neurobehavioral outcomes and stroke severity, further supporting NSE’s clinical relevance in acute neurological injury [50]. Similarly, Lin et al. [51] revealed that elevated NSE is an independent predictor of adverse prognosis in anti-NMDAR encephalitis, a severe autoimmune encephalopathy.

Our ROC curve analyses corroborated these findings, indicating that Netrin-1, NSE, and S100β each possess significant predictive value for short-term death in SAE patients. Kaplan–Meier survival analyses showed leftward curve shifts, indicative of higher mortality, for patients with low Netrin-1 or high NSE/S100β levels, further emphasizing their prognostic significance. More importantly, MEDCALC analysis revealed that combining the three biomarkers yielded superior predictive accuracy for 28-day mortality compared to any single marker alone.

Together, our findings suggest that SAE patients with Netrin-1 < 114.40 ng/mL, NSE > 9.66 ng/mL, and S100β > 0.99 µg/mL face a significantly greater risk of severe brain damage and early mortality. Integrating these markers into routine ICU assessments for septic patients may enhance clinical decision-making, enabling physicians to stratify risk preoperatively and personalize treatment intensity. Quantitative biomarker profiling thus holds promise for improving the early identification and management of high-risk SAE patients.

## Conclusion

This study demonstrated the clinical relevance and prognostic value of serum Netrin-1, NSE, and S100β in patients with SAE. These biomarkers not only reflect the severity of brain injury but also possess predictive utility for short-term mortality, offering novel insight into early risk assessment and potential avenues for SAE prevention.

However, several limitations should be acknowledged. The study had a modest sample size and a limited follow-up duration, with no evaluation of long-term outcomes. This constraint may introduce overfitting in the ROC curve analysis, potentially inflating model performance in the current dataset but reducing generalizability to new data. To address this, cross-validation or external dataset validation is warranted but was not feasible due to time and resource limitations. Future work will involve expanding the cohort and implementing multi-center validation to ensure model robustness. Additionally, while blood samples were collected within 48 h of admission, variations in sampling timing may have affected biomarker levels, particularly given the dynamic expression patterns of inflammatory mediators such as IL-6 and CRP during early sepsis [52, 53]. Future research should adopt more precise timing protocols for biomarker collection to reduce variability.

In conclusion, serum Netrin-1, NSE, and S100β are promising biomarkers for evaluating brain injury and predicting short-term prognosis in SAE. Further studies with larger, diverse populations and refined methodologies are essential to establish their clinical utility and to develop reliable, real-time diagnostic and prognostic tools for SAE management.

## Supplemental data

**Figure S1.**
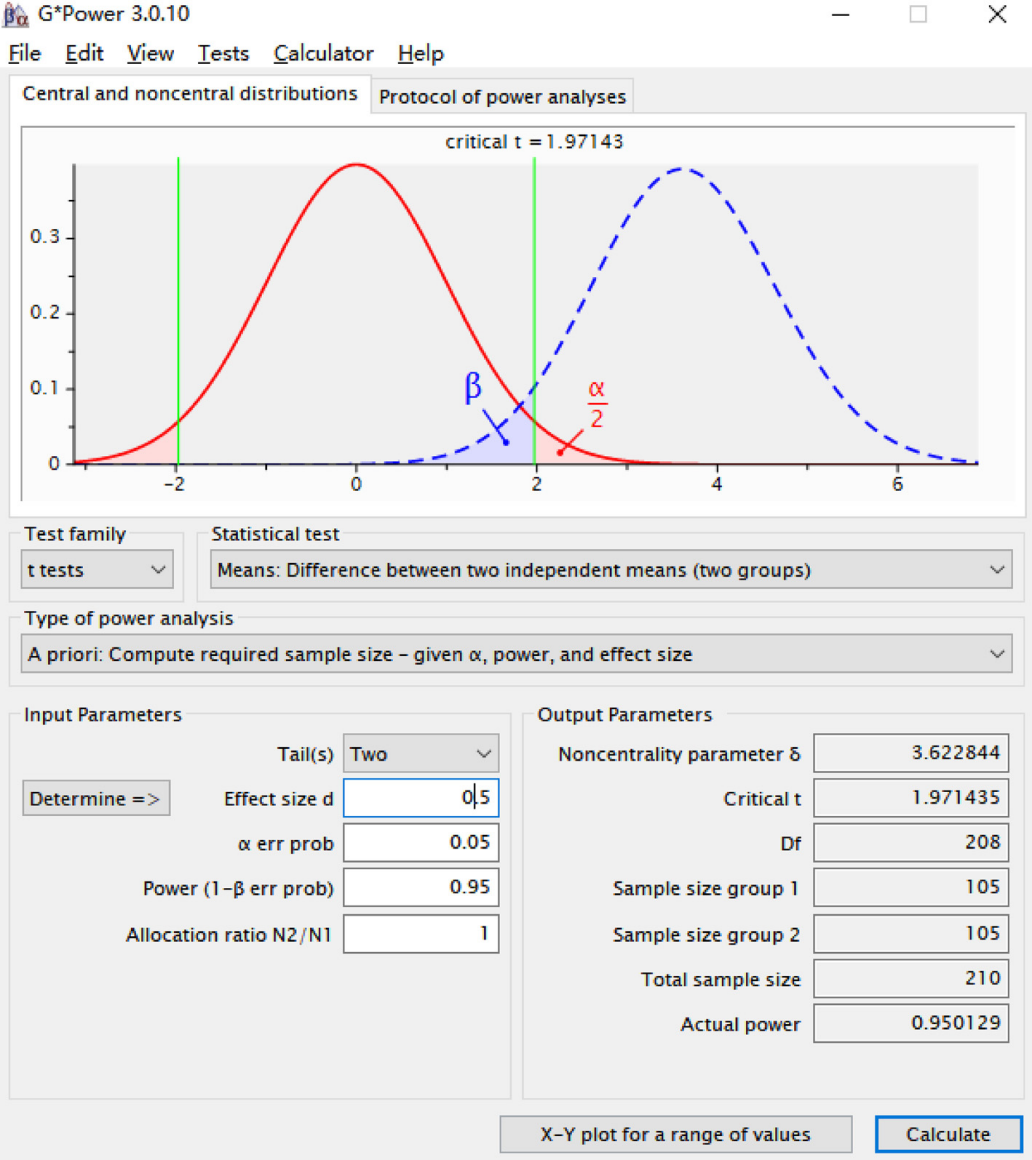
Sample size estimation using G*Power software. Graphical output demonstrating the statistical power and required sample size parameters for the study, calculated using G*Power 3.0.10.

## Data Availability

All data generated or analyzed during this study are included in this article. Further inquiries can be directed to the corresponding author.
